# Note on Æthusa Cynapium

**Published:** 1889-08

**Authors:** E. W. Berridge


### NOTE ON ÆTHUSA CYNAPIUM.

E. W. BERRIDGE, M. D.

At page 263 of vol. X, of Allen's *Encyclopædia*, the author endeavors to discredit the poisonings attributed to this plant, "it having been clearly substantiated that the plant is harmless to produce grave effects." In opposition to this statement, C. Hering calls it (*Guiding Symptoms*, vol. I, p. 74) "A narcotic-acrid poison, on account of an adherent alkaloid substance called Cynapine." Seeing that it has cured "epileptic spasms" (*Guiding Symptoms*), it must be capable of producing them.

I saw in a recent allopathic journal, a case of poisoning by *Æthusa*, the plant being identified by a botanist. Over twenty years ago, at Dr. David Wilson's dispensary, I saw a child cured of sunken cornea with one dose of *Æthusa*$^{1m}$ (Jenichen). I think the symptom is given in Hempel's *Jahr*, under "Post-mortem appearances."

